# Targeting the ERK pathway for the treatment of Cushing's disease

**DOI:** 10.18632/oncotarget.12381

**Published:** 2016-09-30

**Authors:** Dongyun Zhang, Marvin Bergsneider, Marilene B. Wang, Anthony P. Heaney

**Affiliations:** ^1^ Department of Medicine, David Geffen School of Medicine, University of California, Los Angeles, CA, USA; ^2^ Department of Neurosurgery, David Geffen School of Medicine, University of California, Los Angeles, CA, USA; ^3^ Department of Head and Neck Surgery, David Geffen School of Medicine, University of California, Los Angeles, CA, USA

**Keywords:** adrenocorticotropic hormone, Cushing's disease, proopiomelanocortin, testicular receptor 4

## Abstract

We recently demonstrated that the orphan nuclear receptor testicular receptor 4 (TR4) is a potent regulator of corticotroph tumor growth and hormone secretion. The Ras/Raf/MEK/ERK pathway is commonly overactivated in human tumors and we have demonstrated that corticotroph tumor TR4 is activated by ERK1/2-mediated phosphorylation. We evaluated effects of MEK-162, a selective, non-ATP-competitive allosteric inhibitor of MEK1/2, on murine and human *in vitro* and *in vivo* corticotroph tumor proliferation and adrenocorticotrophic hormone (ACTH) secretion. MEK-162 treatment dose-dependently inhibited corticotroph tumor proliferation, induced apoptosis, reduced pro-opiomelanocortin (POMC) mRNA levels and inhibited ACTH secretion *in vitro*. Similar findings were obtained in human corticotroph tumor primary cultures (*n* = 5). These actions of MEK-162 were augmented in the presence of TR4 overexpression, suggesting that TR4 levels may serve as a predictive biomarker of MEK-162 corticotroph tumor responsiveness. Additionally, MEK-162 treatment reduced TR4 protein expression and blocked recruitment of TR4 to bind its consensus site on the POMC promoter (−854bp to −637bp), elucidating multiple mechanisms to control TR4 corticotroph tumor actions. In a murine corticotroph tumor *in vivo* model of Cushing's disease, MEK-162 treatment inhibited tumor growth and reduced tumor-derived circulating plasma ACTH, and corticosterone levels. These results demonstrate the potent actions of MEK-162 to inhibit corticotroph tumor growth and hormone secretion *in vitro* and *in vivo* via TR4-dependent and independent mechanisms, and raise the possibility of MEK-162 as a novel therapy for Cushing's disease.

## INTRODUCTION

Pituitary adenomas are commonly encountered intracranial tumors and cause significant morbidity and mortality due to local compressive effects and hormonal hypersecretion [1]. In particular Cushing's disease, due to an adrenocorticotrophic hormone (ACTH)-secreting pituitary adenoma, results in excessive adrenal cortisol secretion, leading to increased morbidity and mortality [2–5]. Surgical removal *via* transnasal, transphenoidal resection is currently first-line therapy offering initial remission rates of 70-80% in expert centers for microadenomas [1]. However, recurrence rates approach 25% by 20 years and in larger tumors (macroadenomas > 1 cm), initial remission rates are 50% at best. Thereafter, pituitary directed radiation or bilateral adrenalectomy can be offered but both these treatments are associated with significant morbidity. Currently available drugs for Cushing's disease treatment include the dopamine D2 receptor agonist, cabergoline and the somatostatin receptor ligand pasireotide that inhibit tumor-derived ACTH secretion; steroidogenesis inhibitors such as ketoconazole and metyrapone; and the glucocorticoid receptor antagonist mifepristone [1, 6]. However, medical therapies are most commonly used as pre-operative treatment of severe hypercortisolism as side effects or escape from biochemical control otherwise limit long-term use. Safe and efficacious therapies that act directly on the tumor to control both hormonal hypersecretion and pituitary corticotroph tumor growth are needed.

MEK-162 (also known as ARRY162 and Binimetinib) is a second generation oral MEK inhibitor which directly inhibits tumor cell proliferation and induces tumor apoptosis. It shows activity against pancreatic, colorectal and non-small cell lung carcinoma [7]. *In vitro* concentrations inhibiting tumor cell proliferation range from 0.1 μM in fibrosarcoma HT1080 cells to 20 μM in non-small cell lung cancer NCI-H1975 cells [8]. In a non-randomized, open-labeled phase II MEK-162 clinical trial in NRAS melanoma, 20% of patients showed a partial response and MEK-162 was well tolerated with most common toxicities being mild skin or gastrointestinal symptoms [6]. Given that TR4, a potent transcriptional regulator of ACTH secretion, requires MAPK-dependent phosphorylation for activation, we evaluated the effects of MEK-162 on corticotroph tumor cell proliferation and ACTH secretion *in vitro* and *in vivo*.

## RESULTS

### MEK-162 treatment inhibits *in vitro* murine and human corticotroph tumor cell growth and reduces ACTH biosynthesis and secretion

**Figure 1:**
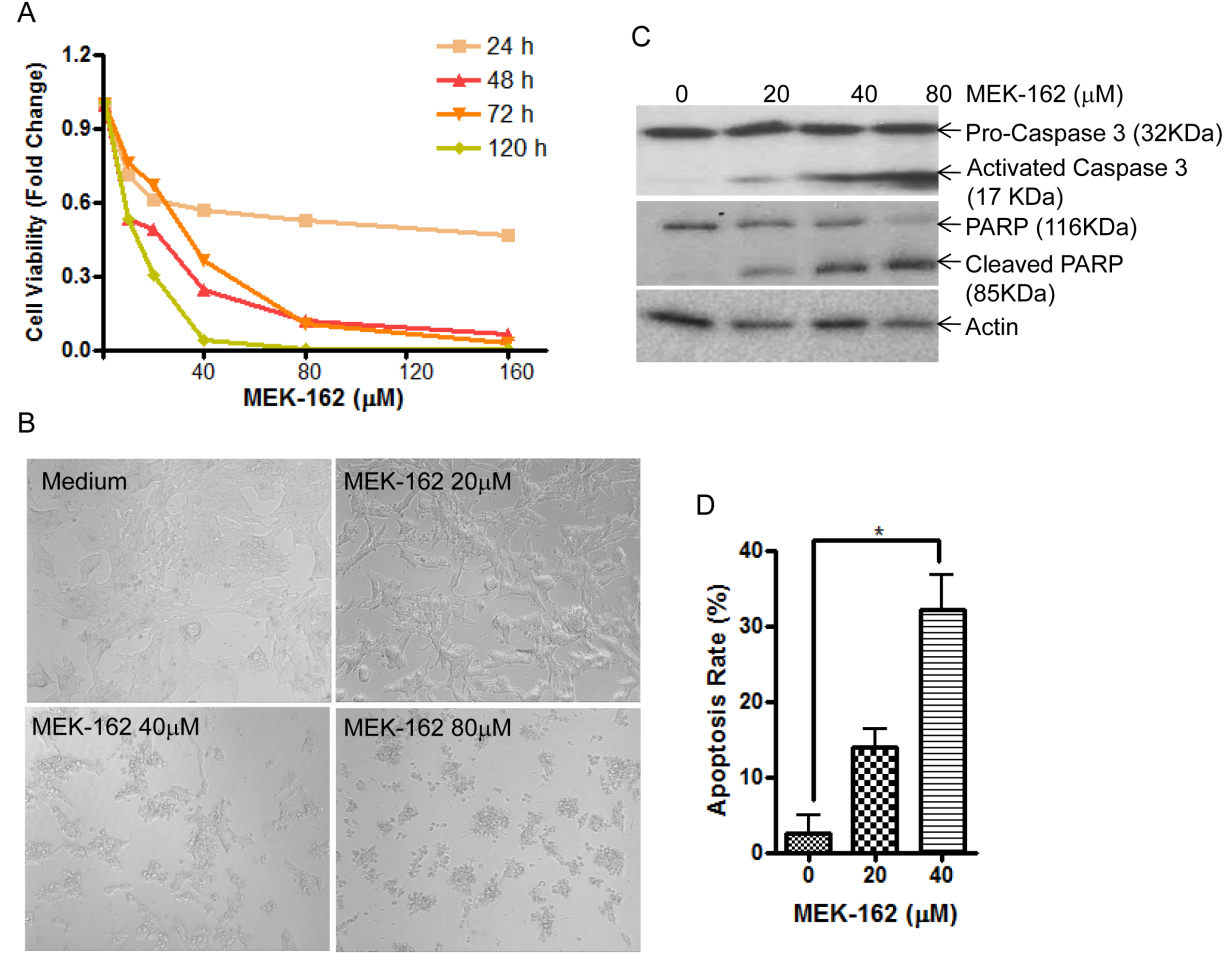
MEK-162 treatment inhibits murine pituitary corticotroph tumor cell proliferation *in vitro* **A.**, Proliferation rates in murine pituitary corticotroph tumor AtT20 cells following MEK-162 treatment (10-160 μM) for 1 to 5 days were analyzed using CellTiter-Glo® luminescent cell viability assay. **B.**, Microscopic changes of AtT20 cells following 48 h MEK-162 treatment showed cell shrinkage and apoptotic bodies. **C.**, Caspase-3 and PARP cleavages, the hall markers of apoptosis, were detected by Western blotting following MEK-162 treatment for 48 h. **D.**, The apoptotic induction rate of MEK-162 treatment for 48 h was detected using a caspase-3 colorimetric assay kit. Each bar indicates the mean ± standard error of triplicate tests. *p < 0.05. Data shown are representative of at least three independently performed experiments.

**Figure 2:**
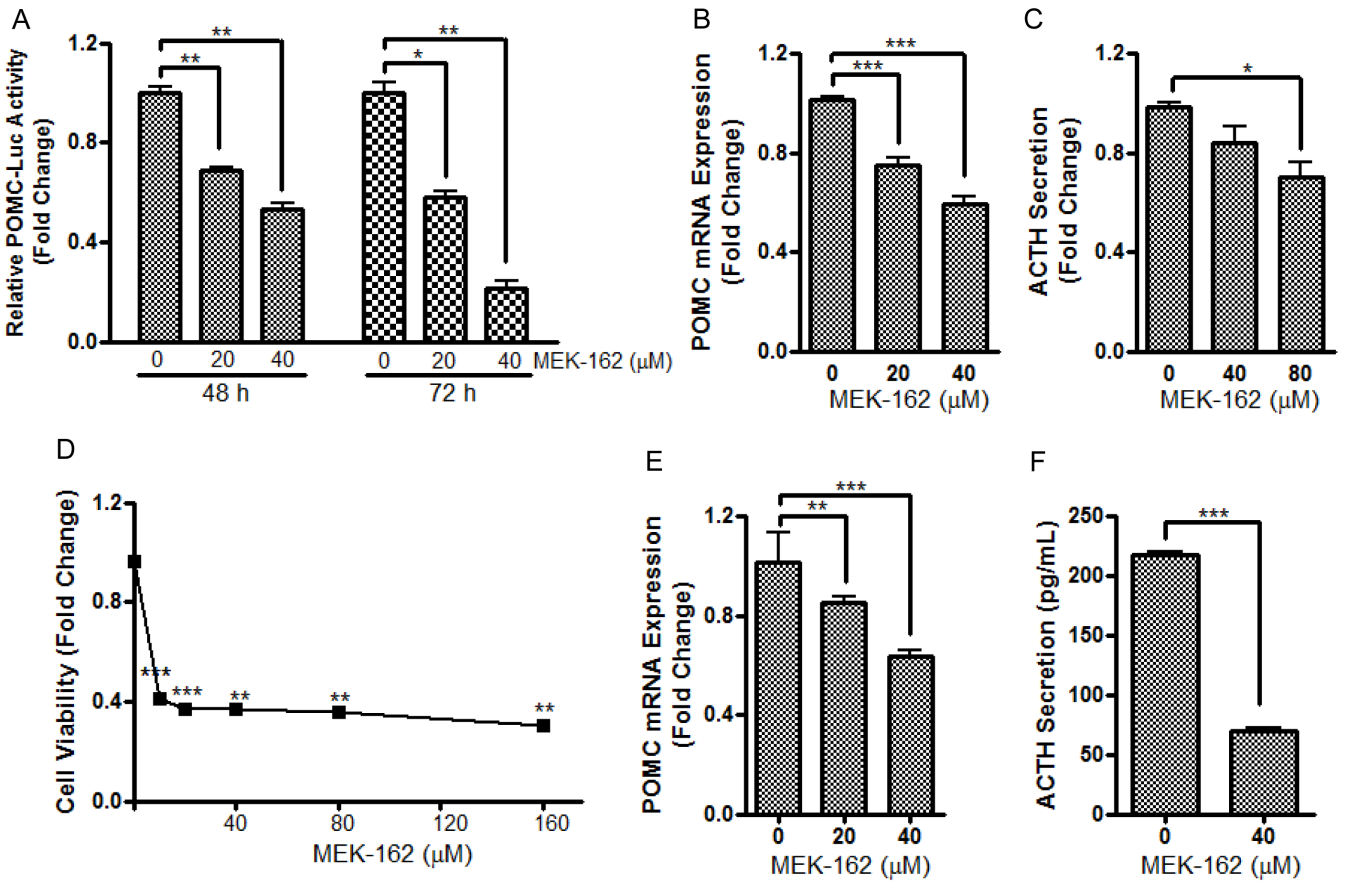
MEK-162 treatment reduces POMC transcription and ACTH secretion in murine and human pituitary corticotroph tumor cells **A.**, AtT20 cells were transfected with a POMC promoter-luciferase reporter. 24 h later, cells were treated with 20 μM and 40 μM MEK-162 in Opti-MEM for 48 and 72 h after which luciferase activities were measured and presented as fold change compared to controls. **B.**, Quantitative PCR (Q-PCR) was used to assess the changes of POMC mRNA following 20 μM and 40 μM MEK-162 treatment for 24 h in Opti-MEM. **C.**, ACTH levels were measured in supernatants from murine AtT20 cells by ELISA assay after 40 μM and 80 μM MEK-162 treatment for 48 h. (D-F), The inhibitory effects of MEK-162 treatment were measured on cell proliferation **D.**, POMC mRNA **E.** and ACTH secretion **F.** in primary culture of freshly resected human corticotroph tumors (*n* = 5). Each bar depicts the mean ± standard error of triplicate tests. * *p* < 0.05; ** *p* < 0.01; *** *p* < 0.005. Data shown are representative of at least three independently performed experiments.

To test potential effects of MEK-162 treatment on *in vitro* corticotroph tumor cell growth and ACTH secretion, murine pituitary corticotroph tumor AtT20 cells were treated with MEK-162 ranging from 10 μM to 160 μM for 1 to 5 days. MEK-162 treatment inhibited corticotroph tumor cell proliferation in a time- and dose-dependent fashion with almost complete inhibition of cell growth at 48 h using higher doses (80 μM and 160 μM, Figure 1A), and ~70% and 93% reduction in cell viability by day 5 with 20 μM and 40 μM MEK-162 respectively (Figure 1A). The MEK-162 treatment induced marked cell shrinkage and membrane-bound apoptotic body formation (Figure 1B). Western blot showed cleavage of Caspase-3 and PARP, key mediators of apoptosis (Figure 1C), and the apoptotic rate was ~30% with 40 μM MEK-162 treatment (Figure 1D). We next examined potential effects of MEK-162 treatment on POMC transcription using AtT20 cells transiently transfected with a POMC-promoter luciferase reporter construct. As depicted in Figure 2A and 2B, MEK-162 treatment inhibited corticotroph tumor POMC-luciferase (Mean ± SE, POMC-luciferase fold difference (FD), 48 h: Vehicle: 1±0.03; MEK-162 20 μM: 0.7±0.01, *p* < 0.01; MEK-162 40 μM: 0.5±0.03, *p* < 0.01; 72 h: Vehicle: 1±0.1; MEK-162 20 μM: 0.6±0.03, *p* < 0.05; MEK-162 40 μM: 0.2±0.04, *p* < 0.01, Figure 2A) and POMC mRNA levels (Mean ± SE, POMC mRNA FD, Vehicle: 1.1±0.002; MEK-162 20 μM: 0.8±0.05, *p* < 0.005; MEK-162 40 μM: 0.6±0.1, *p* < 0.005, Figure 2B) and induced an ~30% suppression of ACTH secretion (Figure 2C). In parallel studies, effects of MEK-162 treatment were evaluated in primary cultures of surgically resected human corticotroph tumors (*n* = 5). Similar to the observed effects in murine corticotroph tumor cells, MEK-162 treatment resulted in a ~60% reduction in cell proliferation (Figure 2D), ~40% decrease in POMC mRNA (Figure 2E) and ~70% decrease in ACTH secretion at 40 μM (Figure 2F). Taken together, these results demonstrate that MEK-162 treatment inhibited both human and murine pituitary corticotroph tumor cell proliferation, POMC transcription and ACTH secretion *in vitro*.

### MEK-162 inhibition of murine pituitary tumor cell proliferation and ACTH biosynthesis are partially mediated by TR4

**Figure 3:**
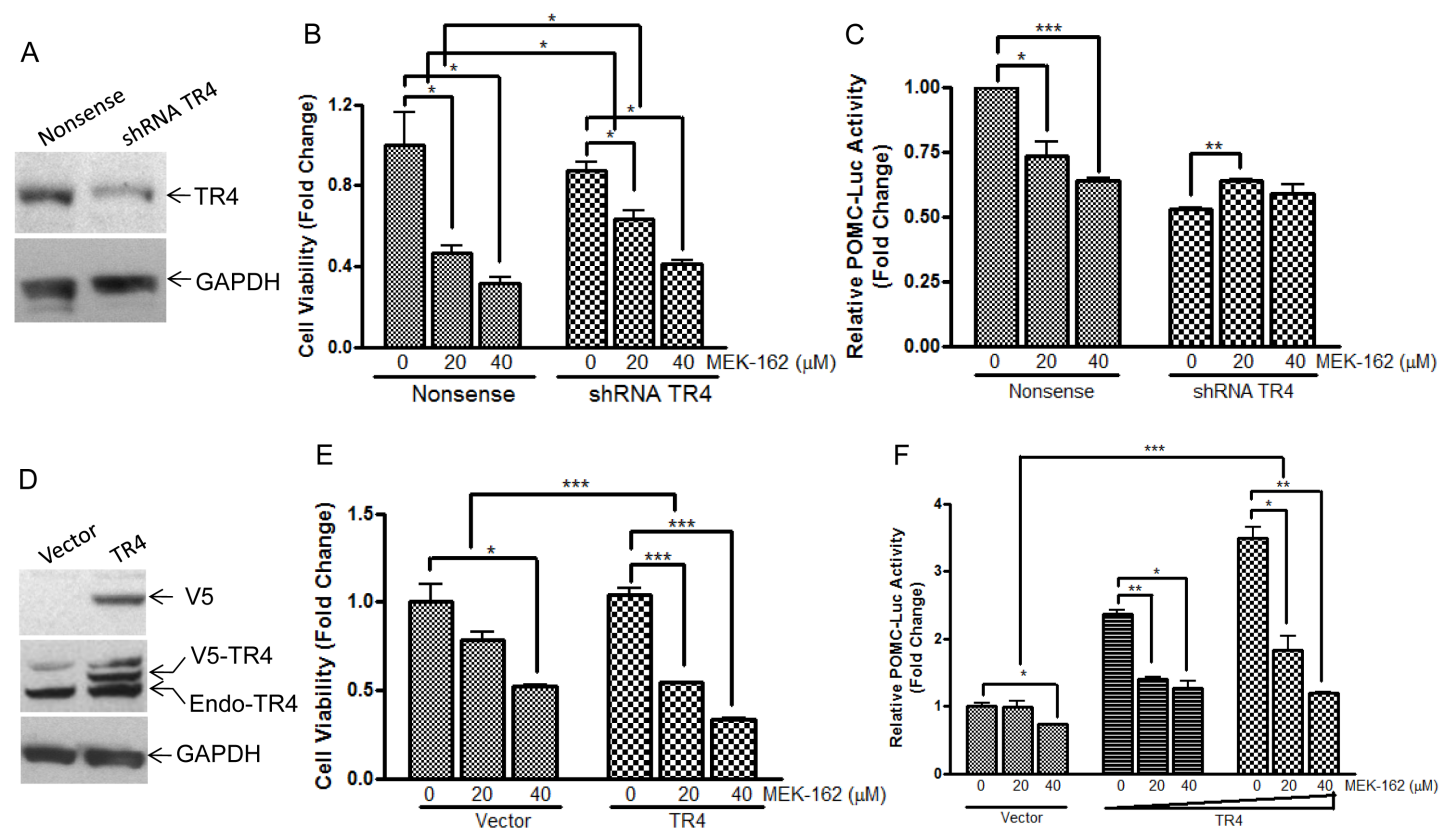

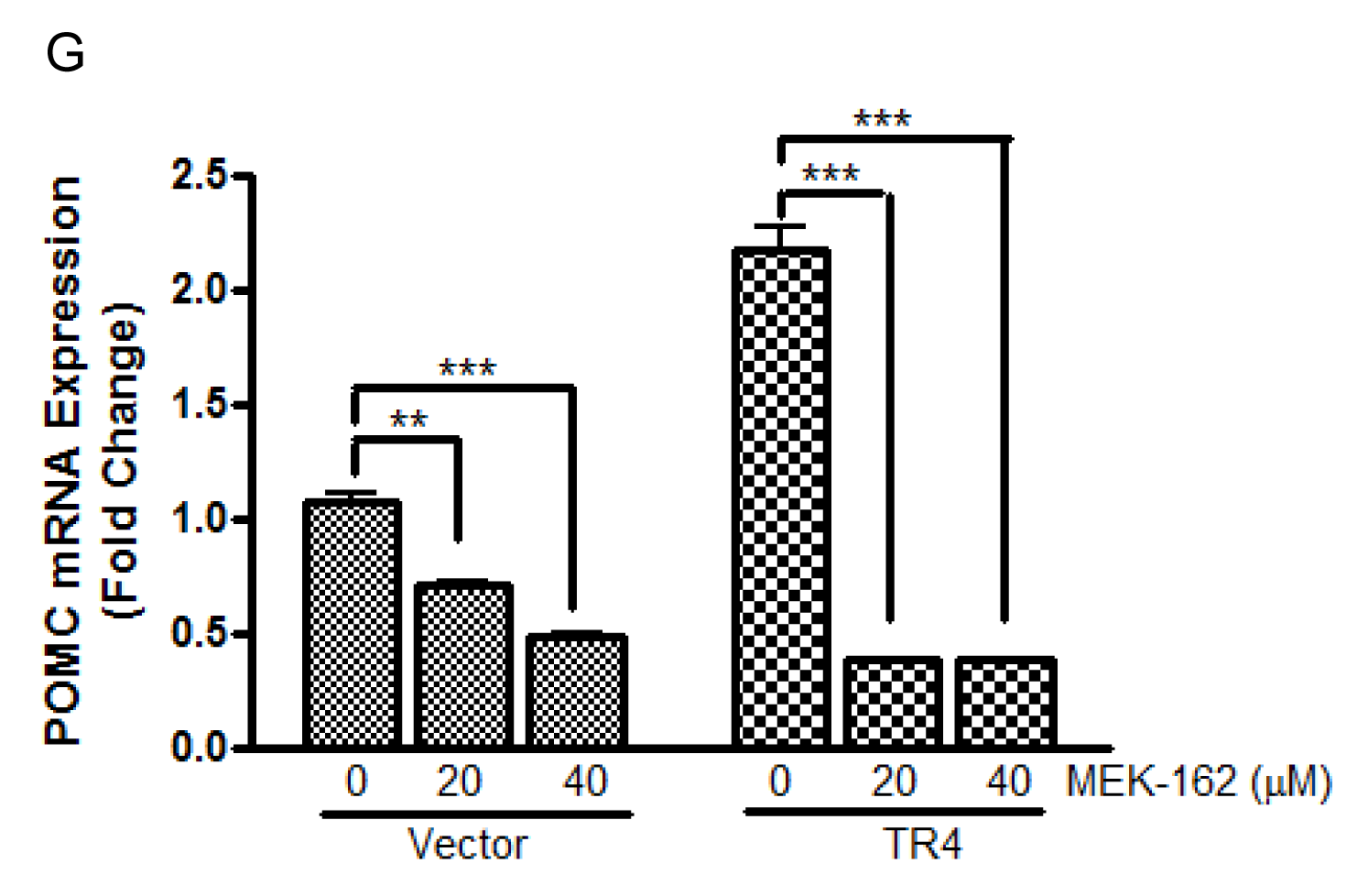
TR4 contributes to the repressive effects of MEK-162 in controlling murine pituitary tumor cell proliferation and hormone synthesis **A.**, Knockdown efficiency of shRNA TR4 in AtT20 cells was evaluated by Western Blotting. **B.**, Proliferation rates in AtT20 shRNA TR4 stable transfectants and nonsense control transfectants were compared following MEK-162 treatment (20 and 40 μM) for 72 h. The results are presented as fold change compared to vehicle treatment in nonsense control transfected cells. **C.**, POMC promoter-luciferase reporter was transiently transfected into AtT20 shRNA TR4 stable transfectants and nonsense control AtT20 transfectants. 24 h later, cells were treated with MEK-162 for 48 h, and luciferase activities were measured and presented as fold change compared to vehicle treatment in nonsense control transfected cells. **D.**, Ectopic expression of V5-tagged TR4 in AtT20 cells was confirmed by Western Blotting. **E.**, AtT20 TR4 stable transfectants and vector control transfectants were treated with MEK-162 (20 and 40 μM) for 72 h to analyze cell proliferation rates. **F.**, POMC promoter-luciferase reporter was transiently co-transfected with TR4 plasmid (200 ng and 400 ng) or vector control into AtT20 cells. 24 h later, cells were treated with MEK-162 in Opti-MEM for 48 h, and luciferase activities were measured and presented as fold change compared to vehicle treatment in vector transfected cells. **G.**, After AtT20 TR4 stable transfectants and vector control transfectants were treated with MEK-162 (20 and 40 μM) for 24 h, POMC mRNA expression was determined by Q-PCR and presented as fold change compared to vehicle treatment in vector transfected cells. All the transfectants used were cell pools. Each bar indicates the mean ± standard error of triplicate tests. * *p* < 0.05; ** *p* < 0.01; ***p < 0.005. Data shown are representative of at least three independently performed experiments.

We recently demonstrated abundant nuclear expression of TR4 in human corticotroph pituitary tumors and demonstrated that active phosphorylated TR4 potently induces POMC transcription as well as murine corticotroph tumor cell proliferation and invasion [9]. To further investigate MEK-162 treatment effects on TR4 corticotroph tumor action, we compared the anti-proliferative effect of MEK-162 treatment in the presence of varying TR4 levels, including knockdown of TR4 by shRNA (Figure 3A) and overexpression of a V5-tagged TR4 construct (Figure 3D). As shown in Figure 3B, whereas MEK-162 treatment (20 and 40 μM for 72 h) inhibited proliferation of corticotroph tumor cells transfected with a nonsense control (Mean ± SE, proliferation FD, Vehicle: 1±0.001; MEK-162 20 μM: 0.4±0.004, *p* < 0.05; MEK-162 40 μM: 0.3±0.02, *p* < 0.05, Figure 3B), knockdown of TR4 attenuated the growth inhibitory effects of MEK-162 treatment (Mean ± SE, proliferation FD, Vehicle: 0.8±0.06; MEK-162 20 μM: 0.6±0.07, *p* < 0.05; MEK-162 40 μM: 0.4±0.03, *p* < 0.05, Figure 3B). In parallel, TR4 knockdown blunted basal corticotroph POMC promoter activity (Mean ± SE, POMC-luciferase FD, Nonsense control: 1±0.001; shRNA TR4: 0.5±0.01, *p* < 0.01, Figure 3C), and addition of MEK-162 did not result in the same inhibitory effects on POMC transcription in shRNA TR4 transfectants (Mean ± SE, POMC-luciferase FD, Vehicle: 0.5±0.01; MEK-162 20 μM: 0.6±0.01, *p* < 0.01; MEK-162 40 μM: 0.6±0.05, Figure 3C) in comparison to MEK-162 effects in nonsense control cells (Mean ± SE, POMC-luciferase FD, Vehicle: 1±0.001; MEK-162 20 μM: 0.7±0.08, *p* < 0.05; MEK-162 40 μM: 0.6±0.01, *p* < 0.005, Figure 3C). In contrast to our finding that reduced TR4 expression blunted MEK-162 actions, TR4 overexpression in murine corticotroph tumor cells augmented the MEK-162 mediated inhibition of cell proliferation (Mean ± SE, proliferation FD, Vehicle: 1.0±0.04; MEK-162 20 μM: 0.5±0.003, *p* < 0.005; MEK-162 40 μM: 0.3±0.004, *p* < 0.005, Figure 3E) compared to vehicle-treated control transfectants (Mean ± SE, proliferation FD, Vehicle: 1.0±0.1; MEK-162 20 μM: 0.8±0.04; MEK-162, 40 μM: 0.5±0.01, *p* < 0.05, Figure 3E). TR4 overexpression also enhanced MEK-162 actions to inhibit POMC transcription compared to vector control transfectants (Mean ± SE, POMC-luciferase FD, Vector control, Vehicle: 1.0±0.05; MEK-162 20 μM: 1.0±0.1; MEK-162 40 μM: 0.7±0.003, *p* < 0.05; TR4 200 ng, Vehicle: 2.4±0.1; MEK-162 20 μM: 1.4±0.03, *p* < 0.01; MEK-162 40 μM: 1.3 ±0.1, *p* < 0.05; TR4 400 ng, Vehicle: 3.5±0.2; MEK-162 20 μM: 1.8±0.2, *p* < 0.05; MEK-162 40 μM: 1.2±0.01, *p* < 0.01, Figure 3F). TR4 overexpression also augmented the actions of MEK-162 to inhibit POMC mRNA expression (Mean ± SE, POMC mRNA FD, Vehicle: 2.2±0.1; MEK-162 20 μM: 0.4±0.002, *p* < 0.005; MEK-162 40 μM: 0.4±0.01, *p* < 0.005, Figure 3G), compared to vector control transfectants (Mean ± SE, POMC mRNA FD, Vehicle: 1.1±0.05; MEK-162 20 μM: 0.7±0.01, *p* < 0.01*;* 40 μM: 0.5±0.01, *p* < 0.005, Figure 3G). These results confirm a role for TR4 in mediating some actions of the MEK pathway on corticotroph tumor proliferation and hormone secretion.

### MEK-162 treatment reduces corticotroph TR4 expression and inhibits its binding to POMC promoter

**Figure 4:**
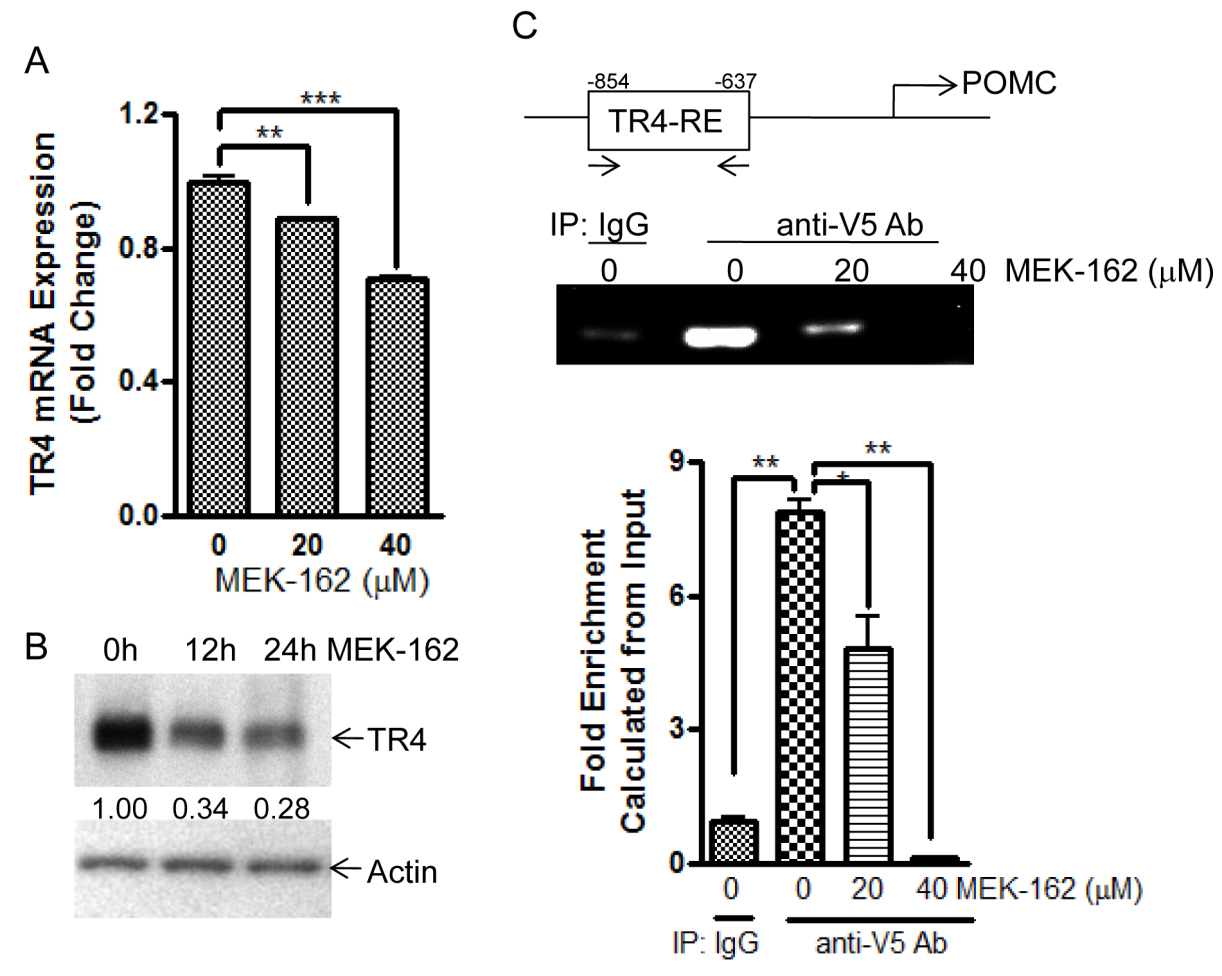
MEK-162 treatment reduces TR4 expression and inhibits binding to POMC promoter **A.**, Q-PCR was used to quantitate TR4 mRNA in murine corticotroph tumor AtT20 cells following MEK-162 treatment (20 μM and 40 μM for 24 h). **B.**, TR4 protein expression was detected by Western Blotting in murine corticotroph tumor AtT20 cells following 40 μM MEK-162 treatments for 12 h and 24 h, and quantified using densitometric analyses of the TR4 protein bands *vs*. the individual loading controls. **C.**, AtT20 cells were transiently transfected with V5-TR4 plasmid. 24 h later, cells were treated with 20 μM and 40 μM MEK for 24 h, after which ChIP assay was performed to measure TR4 binding to the POMC promoter (−854bp~ −637bp). Each bar indicates the mean ± standard error of triplicate tests. * *p* < 0.05; ** *p* < 0.01, *** *p* < 0.005. Data shown were representative of at least three independently repeated experiments.

In some tissues TR4 expression is regulated by activation of the transcription factor C/EBP by a MEK/ERK-dependent pathway [10, 11]. To test if MEK-162 treatment could modulate TR4 action by targeting gene transcription as well as its post-translational modification, we evaluated the effects of MEK-162 on TR4 expression and TR4 binding to the POMC promoter using ChIP assays. MEK-162 treatment of murine corticotroph tumor cells reduced TR4 mRNA (Mean ± SE, TR4 mRNA FD, Vehicle: 1.0±0.01, MEK-162 20 μM: 0.9±0.003, *p* < 0.01; MEK-162 40 μM: 0.7±0.01, *p* < 0.005, Figure 4A). TR4 protein levels reduced by ~30% and ~70% after MEK-162 treatment for 12 h and 24 h respectively (Figure 4B). In ChIP assays MEK-162 treatment reduced the TR4 binding to the POMC promoter (−854bp to −637bp) (Mean ± SE, fold enrichment, Vehicle: 7.9±0.3; MEK-162 20 μM: 4.8±0.7, *p* < 0.05; MEK-162 40 μM: 0.1±0.02, *p* < 0.01, Figure 4C). These results demonstrate that MEK-162 reduces TR4 expression and markedly abrogates the TR4 interaction with the POMC promoter to inhibit corticotroph POMC expression.

### MEK-162 inhibits corticotroph tumor growth, ACTH and corticosterone secretion *in vivo*

**Figure 5:**
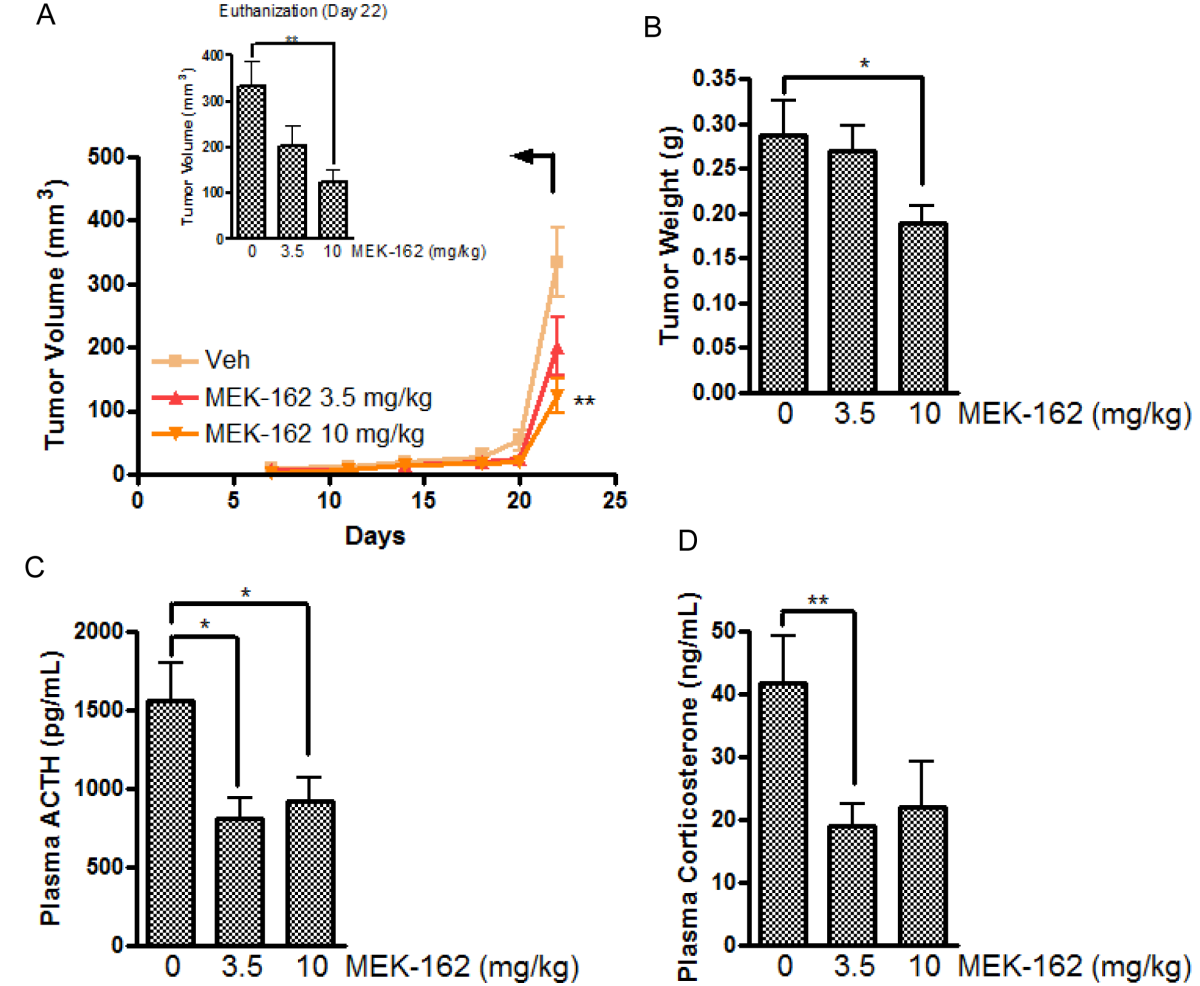
The inhibitory effect of MEK-162 on murine corticotroph tumors *in vivo* Athymic nude mice were inoculated subcutaneously with corticotroph tumor AtT20 cells (5 × 10^5^ cells per mouse in 0.1 mL matrigel). Three days after tumor cell injection, mice were randomly divided into three groups to receive either vehicle or MEK-162 at 3.5 mg· kg−1 or 10 mg ·kg−1 administered p.o. twice a day for 22 days. Graphic depiction of tumor growth rates **A.**, tumor weights **B.**, and circulating ACTH levels **C.** and corticosterone levels **D.** measured in the MEK-treated groups compared to vehicle-treated tumor bearing mice. Values are mean ± standard error, *p < 0.05,**p < 0.01.

We next tested effects of MEK-162 on corticotroph tumor growth, ACTH and corticosterone hormone secretion *in vivo*. Murine corticotroph tumor cells were s.c. inoculated into 5-wk-old male athymic nude mice, and groups of mice were treated with either MEK-162 (3.5 mg· kg^−1^ or 10 mg· kg^−1^) or vehicle twice daily by gavage, starting three days after tumor cell inoculation. At euthanitization (Day 22), tumor volumes (Mean ± SEM, tumor volume mm^3^: Vehicle: 334±55; MEK-162 3.5 mg/kg: 201±47; MEK-162 10 mg/kg: 123±27, *p* < 0.05, Figure 5A) and tumor weights (Mean ± SEM, tumor weights mg: Vehicle: 287±38; MEK-162 3.5 mg/kg: 269±29; MEK-162 10 mg/kg: 189±20 mg, *p <* 0.05, Figure 5B) were lower in MEK-162 treated mice compared to vehicle-treated animals. Consistent with the observed MEK-162-mediated inhibition of tumor growth, decreased plasma ACTH (Mean ± SEM, plasma ACTH pg/ml: Vehicle: 1558±240; MEK-162 3.5 mg/kg: 813±128, *p* < 0.05; MEK-162 10 mg/kg: 919±147, *p* < 0.05, Figure 5C), and corticosterone levels (Mean ± SEM, plasma corticosterone μg/ml: Vehicle: 42±7; MEK-162 3.5 mg/kg: 19±3, *p* < 0.01; MEK-162 10 mg/kg: 22±7, Figure 5D) were seen in MEK-162 treated animals compared to control mice emphasizing the drug's potent inhibition of both corticotroph tumor growth and hormone secretion *in vivo*.

## DISCUSSION

Cushing disease is a life-threatening neuroendocrine disorder caused by a pituitary adenoma, which leads to excess ACTH secretion, and adrenal-derived cortisol. Although surgical resection can offer initial remission in many cases, recurrence rates are high and efficacious medical therapies that control both hormonal hypersecretion and pituitary corticotroph tumor growth with minimal side effects are needed. The Ras/Raf/MEK/ERK pathway is a master regulator of cell proliferation, differentiation and apoptosis [7], and overactivation of this pathway is seen in many tumor types including pituitary tumors [12]. Although the specific targets of Ras/Raf/MEK/ERK pathway in the pathogenesis of pituitary adenomas are largely uncharacterized, our recent findings show that TR4, a potent regulator of corticotroph tumor growth and hormone secretion, is activated by ERK-mediated phosphorylation to regulate hormone synthesis [9]. Therefore, we hypothesized that inhibiting TR4 activation by MEK/ERK pathway blockade would attenuate TR4-mediated corticotroph tumor growth and hormone secretion.

MEK-162 (Array BioPharma Inc.) is a selective non-ATP-competitive MEK inhibitor initially developed as an anti-rheumatoid arthritis drug due to its profound inhibitory effects on the NF-κB pathway leading to decreased IL-1, IL-6 and TNF activities [7]. Its powerful growth inhibitory actions on the MEK/ERK pathway attracted interest in its anti-cancer activity [13]. Human clinical trials of MEK-162 in advanced solid tumors showed an acceptable safety profile and desirable pharmacokinetics properties [14, 15]. In phase II studies of MEK162 in patients with N-Ras and B-Raf mutated advanced melanoma, more than 50% disease control was observed with acceptable side effects, typically rash, diarrhea, fluid retention and creatinine phosphokinase elevation [6]. Given its high tolerability in these clinical trials [6, 14, 15], we examined the effects of this Ras pathway blocker in controlling pituitary corticotroph tumor proliferation and hormone secretion. Our results demonstrate that single reagent MEK-162 treatment exhibits profound dual inhibitory effects on both cell growth and hormone biosynthesis in murine and human corticotroph tumor cells. In addition, the actions of MEK-162 were augmented in the presence of high TR4 expression, suggesting that TR4 might serve as a predictive biomarker of corticotroph tumor responsiveness to MEK-162 treatment. TR4 is an orphan nuclear receptor, and together with another member of the subfamily 2 of nuclear receptors, TR2, play a key role in embryonic development, metabolism and cancer progression [16]. TR4 modulates ATM and GADD45 transcription to regulate DNA repair and cell cycle checkpoints thereby functioning as a genomic guardian to promote cell apoptosis and chromosomal stability [16]. Increasing evidence indicates that several tumor types exhibit increased TR4 expression and support an oncogenic role for TR4 [17]. We have previously shown abundant human corticotroph tumor TR4 expression and that TR4 binds the glucocorticoid receptor to blunt glucocorticoid feedback on corticotroph tumor ACTH secretion [9, 18]. Specifically, we also observed the role of the Ras/Raf/MEK/ERK pathway to phosphorylate and activate TR4, and alter TR4 binding to the POMC promoter [9]. The role of ERK-mediated TR4 activation is all the more relevant given the recent reported finding of USP8 mutation, potentially resulting in increased EGFR action, in ~36% of corticotroph tumor, the latter potentially resulting in increased EGFR action in corticotroph tumors [19].

Our findings here demonstrate that MEK-162 treatment not only reduces TR4 phosphorylation thereby inhibiting its binding to several targets including POMC [9], but simultaneously reduces expression of TR4 itself. These two MEK-162-mediated actions synergistically and potently inhibit TR4-directed actions. These results suggest that MEK-162 may be a novel attractive treatment option for Cushing's disease as it acts directly on the tumor to simultaneously inhibit tumor growth and ACTH secretion. It must be acknowledged that given the many actions of the MEK pathway, a drug such as MEK-162 will inhibit the actions of multiple factors that potentially regulate POMC transcription and will not be limited to TR4. That said, our experiments, in particular the CHIP assay illustrate that some of the actions of MEK-162 in corticotroph tumor cells to inhibit POMC transcription and tumor growth directly involve the actions of TR4 on corticotroph tumors.

## MATERIALS AND METHODS

### Cell culture and reagents

AtT20/D16V-F2 murine corticotroph tumor cells secreting ACTH were purchased from ATCC (CRL-1795) and cultured as monolayer at 37°C, 5% CO_2_ using Dulbecco's Modified Eagles Medium (DMEM, Thermo Fisher Scientific, Hanover Park, IL) containing 10% fetal bovine serum (FBS, Sigma), and penicillin/streptomycin (Thermo Fisher Scientific, Hanover Park, IL). The cultures were detached with trypsin and transferred to new 75-cm^2^ culture flasks twice a week. MEK-162 was provided by Array BioPharma Inc.. For *in vitro* cell culture usage, it was solved in DMSO at the concentration of 100 mM and stored in −80°C freezer in aliquots.

### Primary human corticotroph cultures

Aliquots of fresh surgically resected human ACTH producing tumor tissues were washed in sterile PBS, minced, and digested with DMEM containing 0.5% BSA, 0.35% collagenase, and 0.1% hyaluronidase at 37 °C for 20 min. After centrifugation, cell pellets were resuspended in culture medium for 24 h and then the cells were incubated with vehicle or MEK-162 for measurements of cell proliferation and/or ACTH secretion.

### Plasmid constructs and transfection

The plasmid containing 1kb human POMC promoter fused with luciferase reporter was a gift from Dr. Jacques Drouin (Institute de Recherches Cliniques de Montreal, Montréal, Québec, Canada). Considering the previously reported non-specific effect of TR4 on viral promoter activity [16], protein concentration was used as internal control to normalize firefly luciferase results. ShRNA TR4 construct (V3LMM_499621) was purchased from GE Healthcare (Lafayette, CO). The plasmid V5-TR4 containing mouse TR4 fused to V5 tag was a gift from Dr. Michael Downs and Ronald M. Evans (The Salk Institute for Biological Studies, San Diego, CA). The vector pcDNA3.1/V5-HisB was purchased from Invitrogen (Grand Island, NY). All constructs were verified by sequencing.

### Cell proliferation assay

Parental AtT20 cells, shRNA TR4, TR4 overexpression plasmid and control stable transfectants, and primary cultures of human pituitary corticotrophic ACTH secreting tumors were suspended in 100 μl DMEM supplemented with 10% FBS, and plated in 96-well plates (2×10^3^ viable cells/well) and cultured overnight. Cells were then treated with MEK-162 (10 to 160 μM) for a range of times (1 to 5 days). Cell viability was determined using CellTiter-Glo^®^ Luminescent Cell Viability Assay kit (Promega, Madison, WI) with a luminometer (Wallac 1420 Victor 2 multipliable counter system). Results are presented as proliferation index (relative luminescence signal to medium control) and all experiments were repeated at least three times and shown as medium ± standard error (Mean ± SE).

### Apoptosis assay

For measurement of MEK-162 actions on apoptosis, a Caspase-3 Colorimetric Assay Kit (BioVision, Inc., Milpitas, CA) was utilized. Briefly, following MEK-162 treatment (20 and 40 μM for 48 h), cells were resuspended in 50 μl of chilled cell lysis buffer and incubated on ice for 10 minutes. Supernatant was collected after centrifugation (10,000 × g, 1 min) and protein concentrations were quantified by Nanodrop Lite (Thermo Fisher Scientific Inc. Wilmington, DE). Equal amounts of protein were incubated with 5 μl of the 4 mM DEVD-pNA substrate in reaction buffer (containing 10 mM DTT) at 37°C for 2 hours after which optical densities were read at 405 nm. The fold-increase in Caspase activity was then determined for each group by comparison to the un-induced control. All experiments were repeated at least three times and shown as medium ± standard error.

### Real-time PCR

Total RNA was extracted with RNeasy kit (Qiagen). RNA quantification and integrity were assessed by measurement of absorbance at 260 and 280 nm. Total RNA was reverse transcribed into first-strand cDNA using a cDNA synthesis kit (Invitrogen). Quantitative PCR reactions were carried out using CFX Real-time PCR Detection System (Bio-Rad Laboratories Inc.). Primer sequences (Invitrogen/Life Technologies) were as follows: mouse *β-actin* forward primer, 5′-GGC TGT ATT CCC CTC CAT CG-3′; mouse *β-actin* reverse primer, 5′-CCA GTT GGT AAC AAT GCC ATG T-3′; mouse *POMC* forward primer, 5′- CCA TAG ATG TGT GGA GCT GGT G-3′; mouse *POMC* reverse primer,5′-CAT CTC CGT TGC CAG GAA ACA C-3′; mouse *TR4* forward primer, 5′-GAC TCT GCG GTA GCC TCA C-3′; mouse *TR4* reverse primer, 5′-AGG ATG AAC TGC TGT TTA GAG GA-3′.

### Western blotting

After treatments, cells were washed in cold PBS, and proteins were extracted in 100 μL of radioimmuno precipitation assay (RIPA) buffer (Cell Signaling, Danvers, MA) containing complete protease inhibitor mixture tablets (Roche Molecular Biochemicals, Indianapolis, IN). Protein concentrations were determined by DC protein assay reagent (Bio-Rad, Hercules, CA) and extracts resolved by SDS/PAGE, and transferred to PVDF membranes (Bio-Rad, Hercules, CA). Membranes were blocked for 2 h at room temperature in 0.1% TBS-Tween-20 containing 5% nonfat dried milk, washed, and then incubated with the specific primary antibodies, including anti-Caspase-3 (#9662), -PARP (#9542) (Cell Signaling Technology, Danvers, MA); Anti-Actin (sc-1616) (Santa Cruz Biotechnology Inc., Dallas, Texas); anti-TR4 (ab109301) (Abcam Inc., Cambridge, MA); anti-V5 (R960-25) (Thermo Fisher Scientific, Hanover Park, IL); and anti-GAPH (GTX627408, GeneTex International Corporation). After washing, membranes were incubated with HRP-conjugated secondary antibodies (Santa Cruz Biotechnology Inc., Dallas, Texas) and proteins visualized using a Super Signal Chemiluminescence Assay kit (Pierce, Grand Island, NY). The densitometric analyses of the protein bands *vs*. the individual loading controls were performed using the ImageQuant 5.2 software (GE Healthcare, Pittsburgh, PA). The results shown were representative of three independent experiments.

### Chromatin immunoprecipitation assay

AtT20 cells were transiently transfected with V5-mTR4 construct. Twenty-four hours later, the cells were treated with 20 μM and 40 μM MEK-162 for 24 h. ChIP assay was carried out using EZ-ChIP™ Chromatin Immunoprecipitation Kit (Billerica, MA) and anti-V5 antibody or mouse IgG control (Invitrogen, Grand Island, NY). Co-immunoprecipitated DNA was analyzed by PCR using paired POMC-promoter specific primers (forward 5'-GTA GAT TAG GCA GGC ACC CCG ACT G-3' and reverse 5'-GAA TGG TCT GGG TGG GGA TTG TCT G-3').

### Hormone assays

ELISAs for mouse and human ACTH, and mouse corticosterone were performed in triplicate using reagents purchased from Calbiotech (Spring Valley, AC).

### Tumour xenografts and *in vivo* treatment

The use of mice was approved by the University of California Los Angeles (UCLA) Animal Research Committee (ARC) and complied with all relevant federal guidelines and institutional policies. AtT20 cells (5 × 10^5^) in 100 μL matrigel were injected s.c. into 5-week-old Nu/J (JAX) mice to generate pituitary tumors. Three days after inoculation, mice were randomly divided into three groups (each containing 20 mice), treated with either vehicle or MEK-162 3.5 mg kg−1 or 10 mg kg−1 dissolved in vehicle (0.5% Tween-80, 1% carboxymethyl cellulose) and administered p.o. twice daily [20]. Tumor volumes were measured every other day in two dimensions with Vernier calipers and calculated using the following equation: length × width^2^ ×0.5. Upon completion of MEK-162 treatment, mice were euthanized using CO_2_ inhalation, cardiac blood was collected, and tumors were excised and weighed.

### Statistics

All *in vitro* experiments were repeated at least three times. Results are expressed as mean ± SE. Differences were assessed student *t* test. P values less than 0.05 were considered significant.
